# Biomimetic Mustard Seed-Inspired Brush-Free Alternative for Effective Endoscope Channel Cleaning and Decontamination

**DOI:** 10.3390/biomimetics11080571

**Published:** 2026-08-10

**Authors:** Suk-Dae Lim, Hyun Cho, Sun-Ho Choi, Hong-Gun Kim, Young-Soon Kim

**Affiliations:** 1 The DEUM Co. Ltd., Jeonju 55069, Republic of Korea; powersd@hanmail.net; 2 Department of Biology, University of Washington, Seattle, WA 98404, USA; hyunc456@uw.edu; 3 Department of Carbon Convergence Engineering, Jeonju University, Jeonju 55069, Republic of Korea; rokmwinning2014@jj.ac.kr (S.-H.C.); hkim@jj.ac.kr (H.-G.K.); 4 Institute of Carbon Technology, Jeonju University, Jeonju 55069, Republic of Korea

**Keywords:** carbon ball, mustard seed, activated carbon, carbon fiber, endoscope reprocessing, CFD analysis

## Abstract

Inadequate cleaning of flexible endoscope channels remains a major cause of healthcare-associated infections despite established reprocessing protocols. We developed biomimetic carbon balls inspired by mustard seed surface features as a brush-free adjunct for endoscope channel cleaning, combining mild mechanical action with adsorption-based removal of organic debris. Activated carbon (AC) balls and carbon fiber (CF) balls with diameters of 1.8–3.0 mm were fabricated to fit 2.5–3.5 mm working channels and characterized by TGA, BET, SEM, and EDS. Cleaning performance was evaluated using tomato powder residue and microbiological validation, and computational fluid dynamics was used to assess flow behavior in a 2.7 mm channel containing a 2.6 mm ball under suction. CF balls showed more uniform thermal degradation (8.9% residual mass at 1000 °C) and a ribbed fibrous morphology. In contrast, AC balls exhibited porous, irregular structures with strong adsorption capacity, characterized by high thermal stability (86.4–89.3% residual mass) and pore volumes of 0.087–0.108 cm^3^/g and BET surface areas of 63.00 m^2^/g and 21.65 m^2^/g. After residue exposure, AC surfaces retained more particulate matter, while CF surfaces remained cleaner and promoted more pronounced wall interaction. Microbiological testing showed effective decontamination, with bacterial counts reduced below the detection limit. CFD analysis demonstrated pressure-driven gap flow, elevated local velocity, and vortex-induced wall shear stress sufficient to enhance debris removal without apparent channel damage. These results suggest that mustard seed-inspired carbon balls provide a promising brush-free strategy for endoscope reprocessing by integrating adsorption, hydrodynamic agitation, and gentle scouring to improve cleaning safety and efficacy.

## 1. Introduction

Gastrointestinal (GI) diseases represent a significant global health burden with over 10 million individuals worldwide affected annually [1]. Additionally, more than 20 million GI endoscopies are performed yearly in the United States. Endoscopic techniques, ranging from high-definition chromoendoscopy to magnifying endoscopy, fluorescence endoscopy, and more, are extensively used as the primary method for the diagnosis and management of GI diseases.

Because single-use endoscopes can accumulate high price points, many endoscopic centers have become reliant on reusable endoscopes. Endoscopes are inserted through the mouth or anus of a human body and must be thoroughly cleaned for each patient. Therefore, guidelines for the cleaning and disinfecting procedures for endoscopes have been established [2,3]. These guidelines highlight two parts in cleaning the endoscope: manual cleaning and disinfection. When protocols are followed properly, manual cleaning using the bristle brush, water, detergents, and enzymatic products should remove up to 99.9% of the microbial burden on the endoscope [4,5].

However, manual cleaning is heavily reliant on meticulous human effort. Where even slight deviations from protocol and technique can leave behind microbial residuals, there is great potential for health hazards from inadequate cleaning to fester. Consequently, a study by van der Ploeg et al. revealed that at least 15% of patients on whom endoscopic instruments were used were contaminated with gastrointestinal pathogenic microorganisms [6,7]. Other studies identified the prevalence of the type of infectious bacteria, many of which were identified as Enterobacter cloacae, Escherichia coli, Klebsiella pneumonia and yeasts [8,9,10,11]. This hazard has not escaped the attention of The Emergency Care Research Institute’s “Top Ten Health Technology Hazards,” which points to inadequate manual cleaning as a significant cause of nosocomial infections and iatrogenic transmission [12,13,14] and relates contaminated endoscopes to infectious outbreaks more than to any other reusable medical device [15,16,17].

This present paper relates a carbon ball as an additional safeguard and potential bristle-brush replacement for the hygiene and safety of endoscopic procedures. First, it has been discussed that guideline protocols utilize the bristle brush to manually remove contaminants. Flexible GI endoscopes are easily damaged and can become dented by the incorrect handling of brushes and the presence of bends or other damage on the brushes. Damage to endoscopic channels results in the entrapment of soil and microorganisms within the dents, creating an environment for biofilms to fester [18,19,20].

The design and interfacial mechanics of these carbon balls are directly inspired by biomimetic templates of similarly sized black or brown spherical seeds, particularly mustard (Brassica and Sinapis species) and sweet basil (*Ocimum basilicum*) seeds [21,22,23,24,25,26]. Naturally occurring mustard seeds serve as ideal structural models due to their compact spherical diameter (~1.5–2.5 mm), which quantitatively aligns with target endoscope channels and carbon ball dimensions. Scanning electron microscopy of mustard seed coats reveals structured reticulate microsculpturing characterized by raised epidermal cell boundary ridges on a scale of 10–50 µm. We quantitatively translated these surface topological parameters into the ribbed CF carbon balls via engineered macro-ribs (width: ~200–300 µm) and plate-like micro-features (~5–10 µm). Under fluid suction, these protruding ribs increase surface friction and effective interfacial contact area, undergoing micro-rotational and swirling motion to generate vortex-like scouring along channel walls without causing localized point-loading or scratch damage.

Furthermore, the hydration behavior of seed coats provides a strong physical rationale for the synergistic adsorption and hydrodynamic cleaning dynamics of the carbon balls [21,22,23,24,25,26]. Recent bio-interfacial research demonstrates that seed mucilage rapidly hydrates upon liquid immersion, forming a cellulosic hydrogel composite network embedded with nanofibers that dramatically increases fluid retention and particle-binding capacity [23,25,26]. Zhou et al. established that mucilage hydration establishes a highly adsorptive interfacial envelope capable of capturing and anchoring fine particulate matter under hydrodynamic flow [24,26]. Emulating this mucilage hydration mechanism, the porous AC balls utilize dense micropores (<10 nm) and mesoporous architectures to rapidly adsorb and bind organic residue immediately upon liquid wetting. Crucially, this hydration-inspired adsorption allows the carbon balls to securely sequester detached contaminants on their surface while concurrently undergoing fluid-driven swirling and repeated physical collisions against the luminal walls. Rather than a singular sequential step, this combined action—adsorption due to mucilage hydration and swirling motion by ribbed structures to generate vortex-like scouring—is intended to effectively scour stubborn channel soils while preventing the re-deposition of loosened debris, establishing a robust biomimetic framework that bridges surface topology, fluid dynamics, impact detachment, and adsorption performance [21,22,23,24,25,26].

This study introduces spherical carbon balls—fabricated from activated carbon (AC) and ribbed carbon fiber (CF)—as a brush-free alternative that combines gentle mechanical action with adsorption-based decontamination. Unlike rigid brushes, carbon balls (1.8–3.0 mm diameter) generate vortex-induced scouring through micro-rotational motion and fluid turbulence during suction, maximizing wall contact without point-loading or scratching. AC balls provide high-capacity adsorption via porous structures, while ribbed CF balls enhance mechanical detachment through increased surface friction and effective interfacial contact area. Computational fluid dynamics (CFD) simulations are powerful tools for engineering design and optimization, offering significant advantages over traditional experiments. These techniques can analyze complex CO_2_ adsorption processes to enable industrial scale-up or provide detailed insights into complex thermo-fluid systems in nuclear power plant design [27,28]. CFD excels in performance analysis and design optimization, especially in scenarios where repeated experiments for reproducibility incur high costs and consume significant time. By facilitating early design modifications and rapid iteration, these methods streamline process development and enhance overall efficiency, making them an invaluable asset in engineering research and industrial applications. CFD analysis using ANSYS Fluent/CFX quantitatively validates this mechanism, modeling a 2.6 mm carbon ball in a 2.7 mm channel under 0.4 m/s suction flow (typical endoscope washer conditions). Results demonstrate ΔP ≈ 30 Pa driving force, ΔV ≈ 2 m/s high-velocity gap flow, and wall shear stress exceeding bioburden adhesion thresholds—conditions predicted to dislodge residual contaminants without channel damage.

The objectives of this study are threefold:

(i) Characterize thermal stability (TGA), porosity (Brunauer–Emmett–Teller/Barrett–Joyner–Halenda (BET/BJH)), morphology (SEM), and composition (EDS) of AC and CF carbon balls optimized for endoscope channels.

(ii) Demonstrate adsorption of organic residue (tomato powder simulant) and microbiological sterility (<10 CFU (Colony-Forming Unit)/mL per ISO 15883-5 [29]) after CF ball-assisted cleaning.

(iii) Validate hydrodynamic performance via CFD, elucidating pressure/velocity fields and wall shear stress responsible for effective mechanical cleaning.

By integrating experimental validation with numerical modeling, this work establishes carbon balls as a materials-based solution to the persistent gap between visual cleanliness and microbiological safety in endoscope reprocessing, with potential to replace or augment conventional brushing while protecting channel integrity.

## 2. Materials and Methods

### 2.1. Materials, Formulation and Processing Methods

To ensure structural consistency, high formability, and mass producibility, carbon balls were manufactured using a standardized three-zone production workflow comprising raw material preparation (Zone A), automated injection molding (Zone B), and post-processing/sterilization (Zone C). Prior to formulation, all raw materials were strictly characterized. Activated carbon in powder form (AC) (purity > 99%, average particle size ~4 µm, initial BET surface area ~120 m^2^/g) was used from our own production [30]. Hyosung Advanced Materials (Jeonju, Republic of Korea) provided carbon fiber (CF, fiber diameter about 10 µm, purity > 99%), which we ground to an average length of about 150 µm for use.

The composite material for manufacturing balls with a ribbed structure (CF balls) was prepared by compounding 91 wt% Polypropylenewith 9 wt% carbon fibers. Similarly, spherical balls without a ribbed structure (AC balls) were manufactured by compounding 91 wt% elastomer with 9 wt% activated carbon powder. In Zone A, the raw carbon fibers were precision-cut, milled, and thoroughly dried before being compounded with the elastomer matrix and resin binder. The compounded formulation was subsequently delivered into the molding system using an automated loss-in-weight dosing hopper to guarantee accurate batch-to-batch composition.

In Zone B, the prepared formulation was transferred to a high-precision multi-cavity injection molding machine configured with 16 to 32 spherical cavities tailored to endoscope working channel dimensions (target diameters: 1.8 mm, 2.8 mm, and 3.0 mm). Polymer melt injection was performed at a barrel temperature of 200 °C under a hydraulic injection pressure of 60 MPa. In-mold cooling was maintained at a mold temperature of 50 °C for a hold time of 15 s to achieve uniform structural contraction and eliminate microcrack formation. Solidified carbon balls were extracted continuously using an automated robotic pick-and-place system.

In Zone C, post-processing was executed to refine surface geometry and ensure biological safety. The molded balls underwent gate/runner cutting followed by surface finishing (burr removal and micro-sanding) to eliminate localized points of friction. To ensure surface cleanliness, the spheres were subjected to dual-action cleaning comprising ultrasonic bath washing followed by hot-air drying. Quality control (QC) was conducted via automated vision inspection to verify dimensional tolerances and structural integrity. Finally, the carbon balls were subjected to Ethylene Oxide (EO) gas sterilization and sealed in medical-grade packaging to maintain sterility prior to clinical use.

### 2.2. Ball Design and Characterization

**Figure 1:**
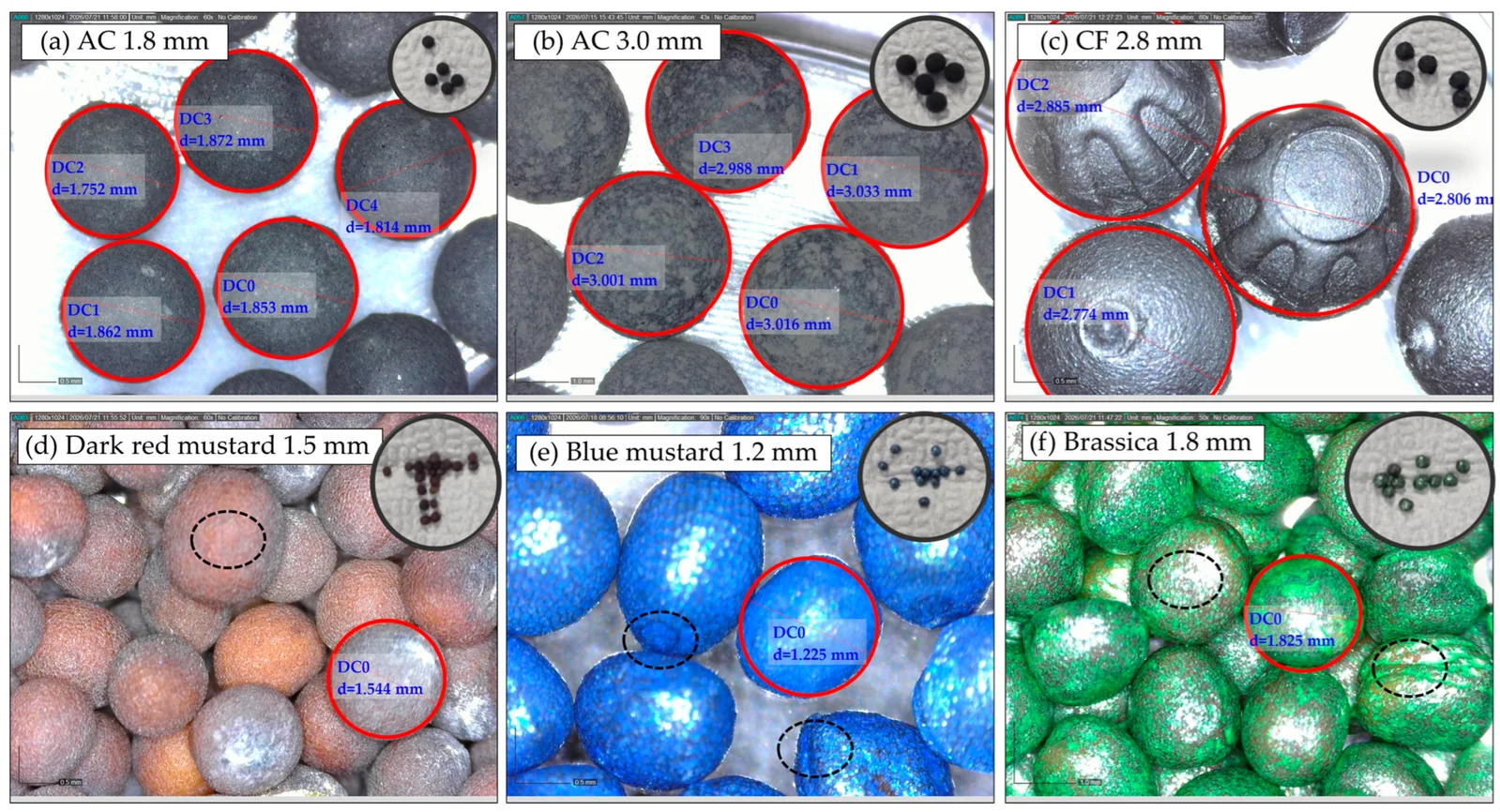
High-resolution digital microscope images confirming the quantitative dimensions and biomimetic design origins of the carbon balls. Calibrated measurements obtained via Dino-Lite AM4113TL display exact spherical diameters for: (**a**) smooth AC balls (1.8 mm), (**b**) smooth AC balls (3.0 mm), and (**c**) ribbed CF balls (2.8 mm). Comparative biological seeds are shown in the bottom row with black dashed circles highlighting particular biomimetic structural inspirations: (**d**) a dark red mustard seed (1.5 mm) with a net-like surface texture resembling the micro-roughness of the AC and CF balls; (**e**) a blue mustard seed (1.2 mm) with a stem-like protrusion and groove that inspired the polar protrusion and macro-ribbed structure on the CF ball; and (**f**) a Brassica seed (1.8 mm) with distinct surface distribution and grooves similar to the spread surface of the AC ball and macro-ribbed structure of the CF ball.

The carbon balls’ quantitative dimensions and biomimetic design origins are confirmed by high-resolution digital microscope photographs in Figure 1. Dino-Lite AM4113TL (AnMo Electronics Corporation, New Taipei City, Taiwan)-calibrated measurements show precise spherical diameters for: (a) smooth AC balls (target: 1.8 mm; measured: 1.752–1.872 mm); (b) smooth AC balls (target: 3.0 mm; measured: 2.988–3.033 mm); and (c) ribbed CF balls (target: 2.8 mm; measured: 2.774–2.885 mm) with surface friction contours. Comparative biological seeds with microscale biomimetic surface topographies are displayed in: (d) Brassica seed (1.825 mm), (e) blue mustard seed (1.225 mm), and (f) dark red mustard seed (1.544 mm). Macroscopic views of the corresponding bulk spheres and seeds are displayed in the insets.

The structural design of the 2.8 mm CF balls was directly inspired by microscale natural biomimicry. As shown in Figure 1d–f, biological seeds such as Brassica (1.8 mm) and mustard species (1.2–1.5 mm) possess intrinsic surface ribs and micro-textured bulges that optimize fluid interactions and mechanical grip. By replicating these seed-inspired topographical ribs onto the CF carbon balls, localized vortex shedding and fluid-driven scouring were significantly enhanced within the endoscope channel lumen. The structural design of the carbon balls was directly inspired by the morphological diversity of natural plant seeds, bridging biological topographies with mechanical functionality. As illustrated in Figure 1, specific anatomical features of natural seeds were mapped and physically replicated onto the engineered carbon spheres.

Detailed observation of the biological seeds (highlighted by black dashed circles in Figure 1d–f) reveals distinct biomimetic correlations that were physically replicated onto the engineered carbon spheres. Specifically, the net-like surface morphology of the dark red mustard seed (Figure 1d) was adopted to create the inherent micro-surface roughness present across all carbon composite balls (Figure 1a–c), maximizing the localized frictional contact area. Additionally, the distinct stem-like protrusion found on the blue mustard seed (Figure 1e) inspired the protruding polar geometry of the CF ball (Figure 1c), which acts as a primary focal point for flow disruption. This design is further complemented by the prominent ridges and grooves on the Brassica seed (Figure 1f), which were translated into the macro-ribbed contours of the CF ball to induce significant fluid turbulence and high wall shear stress during channel transport. Ultimately, the comprehensive surface morphologies of all fabricated AC and CF spheres closely mirror the natural irregular textures of these biological seeds, demonstrating a successful translation of microscale biomimicry for enhanced medical device decontamination.

**Figure 2:**
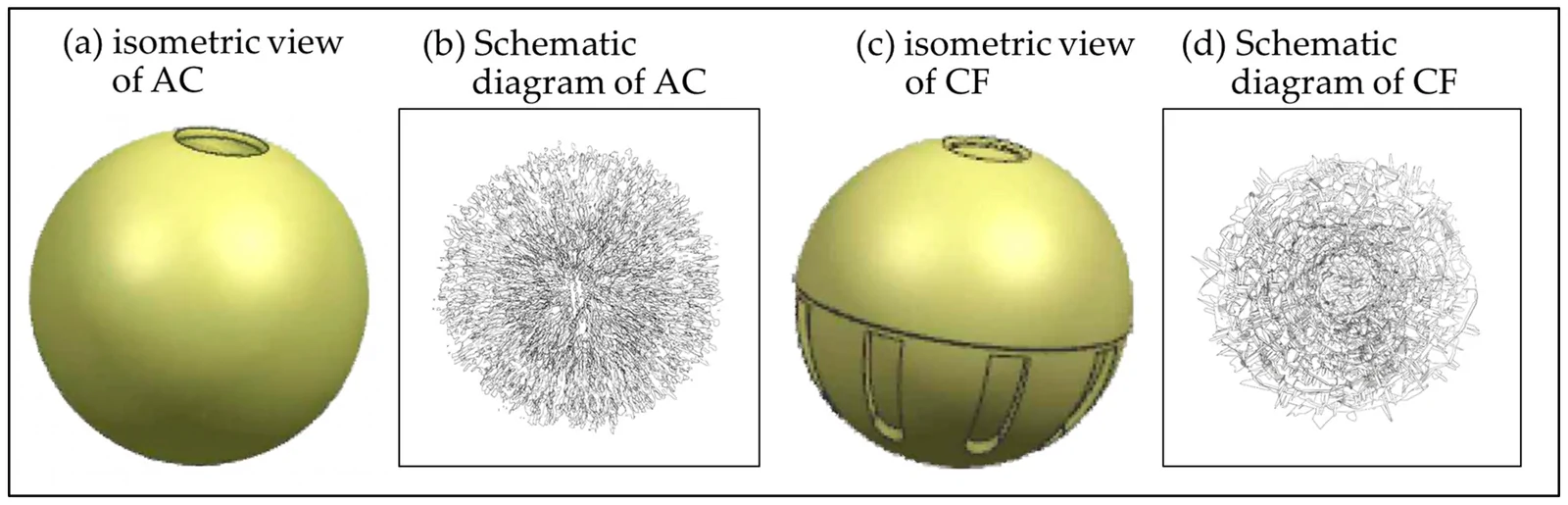
Comprehensive ball design and structural characterization combining 3D CAD modeling, internal network schematics, and optical digital micro-photographs. (**a**) 3D CAD isometric view of the smooth activated carbon (AC) ball. (**b**) Schematic diagram illustrating the internal highly porous network of the AC composite. (**c**) 3D CAD isometric view of the ribbed carbon fiber (CF) ball showcasing friction-inducing surface contours. (**d**) Schematic diagram illustrating the interconnected internal fibrous matrix of the CF composite.

Figure 2a,c are isometric views of Figure 1a–c. Figure 2a is an isometric view of the spherical AC ball from Figure 1a,b and Figure 2c is an isometric view of the CF ball having a ribbed structure in Figure 1c. Figure 2b,d are the schematic diagrams. The AC balls are of a more disordered and irregular composition that resembles a chaotic fluff, with a net-like structure and irregular aggregates, as shown in Figure 2b. This is consistent with the surface morphology shown in Figure 1d,f. In contrast, the CF balls show a composition that is highly ordered and more compact in Figure 2d. This also originates from the polar and valley-like surface morphology shown in Figure 1e,f. Furthermore, Figure 2b,d also demonstrate structural differences that cause the morphological differences visible in the SEM image of Section 3.3. Conceptually, the ordered fibrous architecture of the CF balls resembles interconnected flow pathways that maintain open access to the surface, whereas the disordered AC network can create dead-end pores that are less accessible to larger molecules, as further discussed for heterogeneous activated carbons.

This structural contrast underpins the proposed division of roles during cleaning: AC balls primarily provide volumetric adsorption of released contaminants, while CF balls contribute vortex-induced detachment. In particular, black or brown spherical seeds with a diameter of about 2 mm, such as the seeds of mustard and brassica species, are mainly used as food [31,32,33,34,35]. Accordingly, these are suitable biomimetic templates because their seed coats present structured microsculpturing and, in some species, hydration-induced mucilage that alters interfacial behavior [21,22,23,24,25,26]. Basil seeds can be added as a second model because their mucilage-rich testa provides a natural example of surface-mediated fluid retention and particle stabilization. Together, these seeds support a biomimetics narrative connecting surface morphology, swirling motion response, and adsorption–desorption performance. Among candidate seeds, Brassica and mustard seeds are particularly suitable biomimetic templates because their seed coats exhibit well-defined microsculpturing visible by SEM, while basil seeds provide an additional model owing to their rapid mucilage formation after hydration [26]. These features make them relevant for studying impact-induced detachment, surface adsorption, and post-vortex retention in the 2 mm size range.

### 2.3. Thermogravimetric Analysis (TGA)

Thermal properties of AC 1.8 mm, AC 3.0 mm and CF 2.8 mm balls were assessed by thermogravimetric analysis. Approximately 3.4–4.1 mg of each sample was placed in an alumina pan and heated from 25 to 1000 °C over 10 min in a nitrogen atmosphere using a TGA instrument [Mettler-Toledo, TGA/DSC1, Columbus, OH, USA]. The mass loss profiles and residual weights obtained from TGA were used to evaluate compositional uniformity and to determine the relative thermal stability of each sample type.

### 2.4. Surface Area and Porosity (BET and BJH Analyses)

Nitrogen adsorption–desorption isotherms were recorded at 77 K using a surface area and porosity analyzer [Micromeritics (ASAP 2420, Norcross, GA, USA)]. Surface area and pore characteristics were analyzed using nitrogen (N_2_) adsorption–desorption isotherms. The instrument settings included an analysis bath temperature of −195.85 °C and an equilibration interval of 10 s, with an ambient temperature of 22 °C. The BET measurements were conducted as a single analytical run (*n* = 1) for each sample (0.0409 g for AC 1.8; 0.1523 g for AC 3.0) to characterize baseline material properties; therefore, no statistical treatment was performed on this data. Prior to analysis, all samples were degassed under continuous vacuum at 150 °C for 6 h to remove adsorbed moisture and volatile contaminants. The Brunauer–Emmett–Teller (BET) method was applied to determine specific surface area within a relative pressure (P/P_0_) range of 0.05–0.30, and full adsorption–desorption isotherms were collected over P/P_0_ = 0.01–0.99. Pore size distributions and cumulative pore volumes were derived from the desorption branch using the Barrett–Joyner–Halenda (BJH) method.

### 2.5. Morphological Observation (SEM)

Field-emission scanning electron microscopy (SEM, HITACHI, SU7000, Tokyo, Japan) was performed to examine the surface morphologies. Prior to imaging, samples were mounted on aluminum stubs using conductive carbon tape and sputter-coated with platinum (Pt) (10 mA, 120 s) to prevent surface charging. FE-SEM imaging was conducted at an accelerating voltage of 10 kV and a working distance of 8–10 mm, using a secondary electron detector. Microstructural features including surface roughness, fiber alignment, and particle adhesion were examined across multiple magnifications (122×, 1.00 k×, 5.00 k×, and 50.0 k–100 k×). For each sample type, at least three independent spheres (*n* = 3) were observed across multiple randomly selected regions to ensure structural representativeness.

### 2.6. Energy-Dispersive X-Ray Spectroscopy (EDS)

Elemental mapping and quantitative compositional analyses were conducted using an energy-dispersive X-ray spectrometer (EDS, Oxford Instruments, Abingdon, UK) coupled to the FE-SEM system. EDS spectra and spatial maps were acquired at an accelerating voltage of 15 kV, a beam current of 10 µA, a working distance of 10 mm, and an acquisition live time of 60 s per region over an energy range of 0–20 keV. Quantitative elemental compositions (atomic % and weight %) were extracted after standard matrix correction. Measurements were performed on at least three randomly selected surface regions (*n* = 3) per sample group before and after immersion testing, and the resulting elemental percentages are expressed as means.

### 2.7. Numerical Analysis of Flow and Mechanical Cleaning in Flexible Endoscope Channels

To complement the experimental characterization and cleaning tests, a numerical fluid–structure interaction analysis was performed to evaluate how carbon balls interact with flexible endoscope channel walls and residual bioburden under clinically relevant flow conditions.

#### 2.7.1. Physical Conditions, Boundary Conditions, and Numerical Schemes

**Figure 3:**
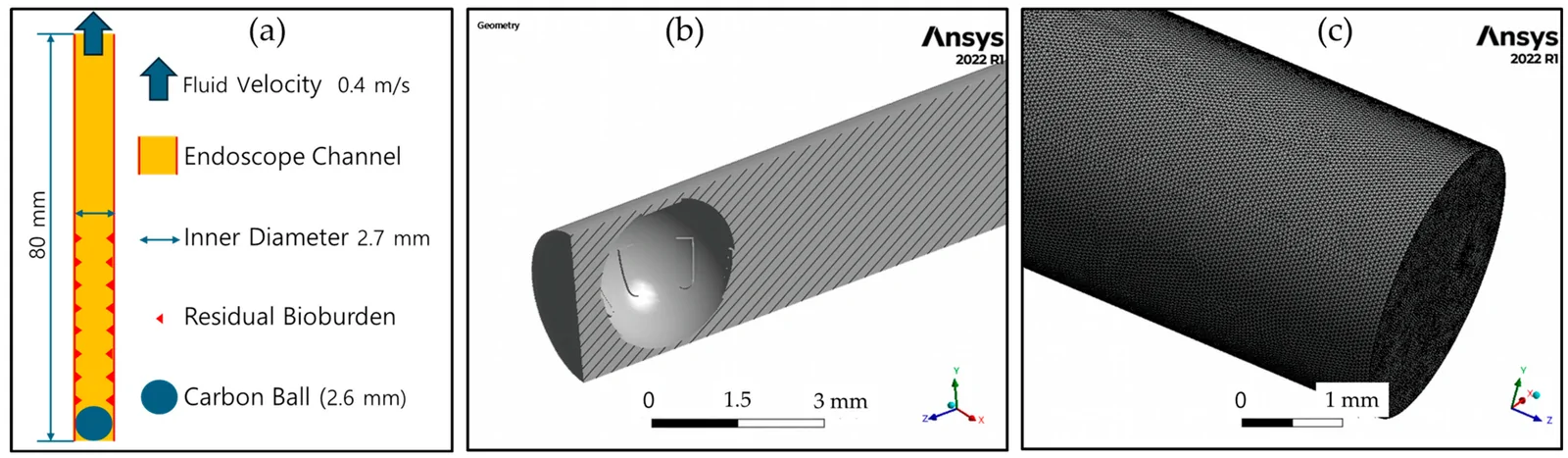
CFD simulation setup and physical conditions: (**a**) schematic diagram, (**b**) cross-sectional geometry, and (**c**) isometric mesh view. An 80 mm long endoscope channel (inner diameter: 2.7 mm) contains a 2.6 mm cleaning ball, leaving a 0.05 mm clearance. Flow is modeled with a suction velocity of 0.4 m/s under fluid property conditions. The ANSYS simulation evaluates flow dynamics (velocity, pressure, vortex, wall shear stress) to assess bioburden removal, where cleaning occurs when wall shear stress exceeds particle adhesion force.

Figure 3 shows the schematic diagram (Figure 3a) and geometrical configuration (Figure 3b,c) of the flexible endoscope channel and carbon ball. A simplified straight working channel model with an inner diameter of 2.7 mm and a length of at least 50 mm was constructed to represent a typical gastrointestinal endoscope channel. A spherical carbon ball with a diameter of 2.6 mm was placed near the center of the lumen, providing a radial clearance of approximately 0.05 mm between the ball surface and the channel wall. This narrow gap was selected to reproduce the conditions under which the ball can closely contact the channel wall while still allowing sufficient fluid flow for mechanical cleaning. A three-dimensional CFD model was developed using ANSYS Fluent/CFX [2022 R1] (Ansys, Inc., Canonsburg, PA, USA). To accurately capture the flow dynamics and the high shear gradients in the narrow gap, the shear stress (k-omega) turbulence model was employed. This model was selected for its robust performance in resolving boundary layers and predicting flow behavior under adverse pressure gradients. At the channel inlet, a suction-type boundary condition with a mean fluid velocity of 0.4 m/s was applied, corresponding to a typical cleaning solution flow rate in automated endoscope reprocessors. Water for cleaning solution was selected as the working fluid, and the density and viscosity of the chosen fluid were used in the simulations. The carbon ball was treated as a stationary body for steady-state simulations, while in transient simulations, the ball was allowed to move along the channel, and its position was updated over time based on the hydrodynamic forces. No-slip boundary conditions were applied at all solid surfaces, including the channel wall and the ball surface, and the outlet was set to a constant reference pressure.

Strict convergence criteria were enforced to ensure numerical stability and accuracy across all simulations. The solution was deemed converged when the scaled residuals for the continuity and momentum equations fell below 10^−5^. Furthermore, surface monitors for the average wall shear stress and the drag force on the carbon ball were tracked continuously during iterations. Computations were considered fully converged only when these monitored variables reached a steady state, exhibiting oscillations of less than 1% over 100 consecutive iterations.

Post-processing focused on four key field variables: velocity distribution, pressure distribution, vorticity, and wall shear stress. The driving force acting on the carbon ball was estimated from the pressure difference between the upstream and downstream sides of the ball, while the mechanical cleaning potential was evaluated from the wall shear stress acting on the bioburden layer. Mechanical removal of residual bioburden was inferred in regions where the local wall shear stress exceeded a critical adhesion threshold reported in recent detachment studies [36,37,38,39]. Specifically, effective mechanical cleaning zones were identified where the imposed flow conditions overcame the structural resistance of the bioburden (Reynolds number from 3.75 to 15, maximum wall shear stress from 10.7 to 42.7 Pa, and shear rate from 2000 to 8000 s^−1^ for a duration of 40 ms) as established in relevant coupled modeling frameworks [39].

#### 2.7.2. Mesh Generation, Independence, and Validation

**Figure 4:**
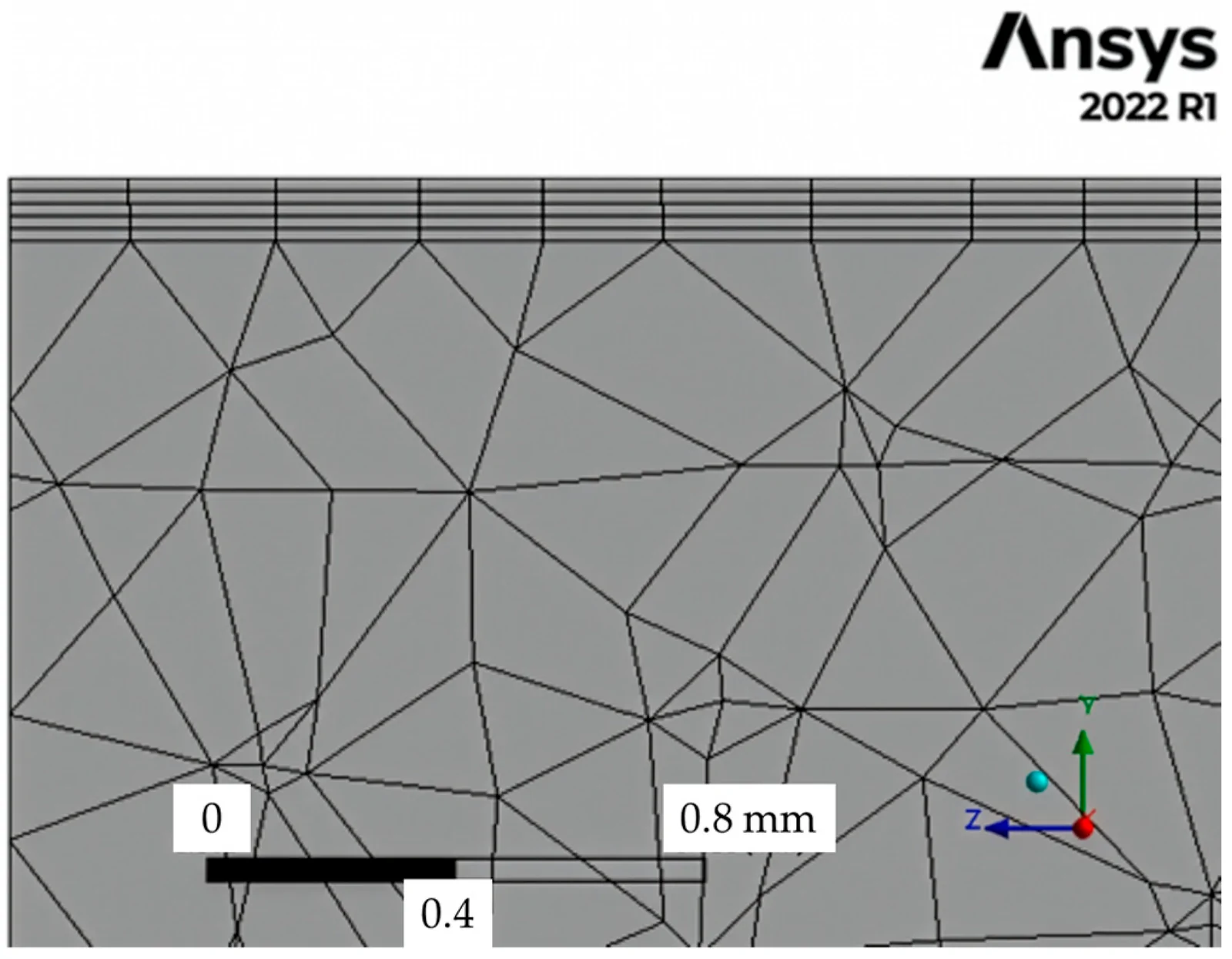
Detailed view of the computational mesh structure showing boundary layer inflation along the pipe wall. The domain incorporates a global pipe mesh size of 0.1 mm, ball edge sizing of 0.05 mm, and 5 inflation layers at the wall to resolve boundary layer dynamics and ensure numerical convergence. The final model consists of 28,134,508 elements and 5,681,067 nodes.

Because the clearance between the channel and the carbon ball was very small, a locally refined mesh was generated in the gap region to capture steep gradients in velocity and wall shear stress. A global mesh size of 0.1 mm was set for the channel, while an edge sizing of 0.05 mm was applied to the carbon ball to ensure detailed geometric resolution. To accurately resolve near-wall flow behavior and guarantee numerical convergence, 5 inflation layers were applied along the channel wall and ball surface, with the first grid cell adjacent to the wall sized to maintain a non-dimensional wall distance (y+) of approximately 1. In addition, a thin residual bioburden layer was defined as a separate region on the inner surface of the channel to enable detailed evaluation of wall shear stress acting on that layer. The potential for removing these residual foreign substances was determined based on the criterion that successful removal is achieved when the localized wall shear stress exceeds a specific critical threshold. The final computational mesh used in the analysis comprised 28,134,508 elements and 5,681,067 nodes. Figure 4 shows the mesh structure used in the analysis, highlighting the fine elements in the narrow gap between the ball and the channel wall.

To ensure the numerical results were independent of grid resolution, a rigorous mesh independence study was conducted. Three distinct mesh densities (coarse, medium, and fine) were evaluated by comparing the maximum wall shear stress in the gap region and the pressure drop across the carbon ball. The medium mesh was ultimately selected for all subsequent analyses, as the variation in key evaluation parameters between the medium and fine meshes was less than 2%, offering an optimal balance between computational accuracy and efficiency.

To validate the computational setup, baseline simulations were benchmarked against established theoretical and empirical data. The pressure drop across the straight channel without the carbon ball was simulated and compared to theoretical values derived from the Hagen–Poiseuille equation for fully developed flow. Additionally, the calculated hydrodynamic drag force on the carbon ball was compared against empirical drag correlations for a sphere in a confined tube. The maximum deviation between the CFD predictions and the benchmark values was maintained within an acceptable margin of 5%, confirming the reliability of the numerical modeling approach for this study.

## 3. Results

### 3.1. Thermal Behavior of Carbon Balls

**Figure 5:**
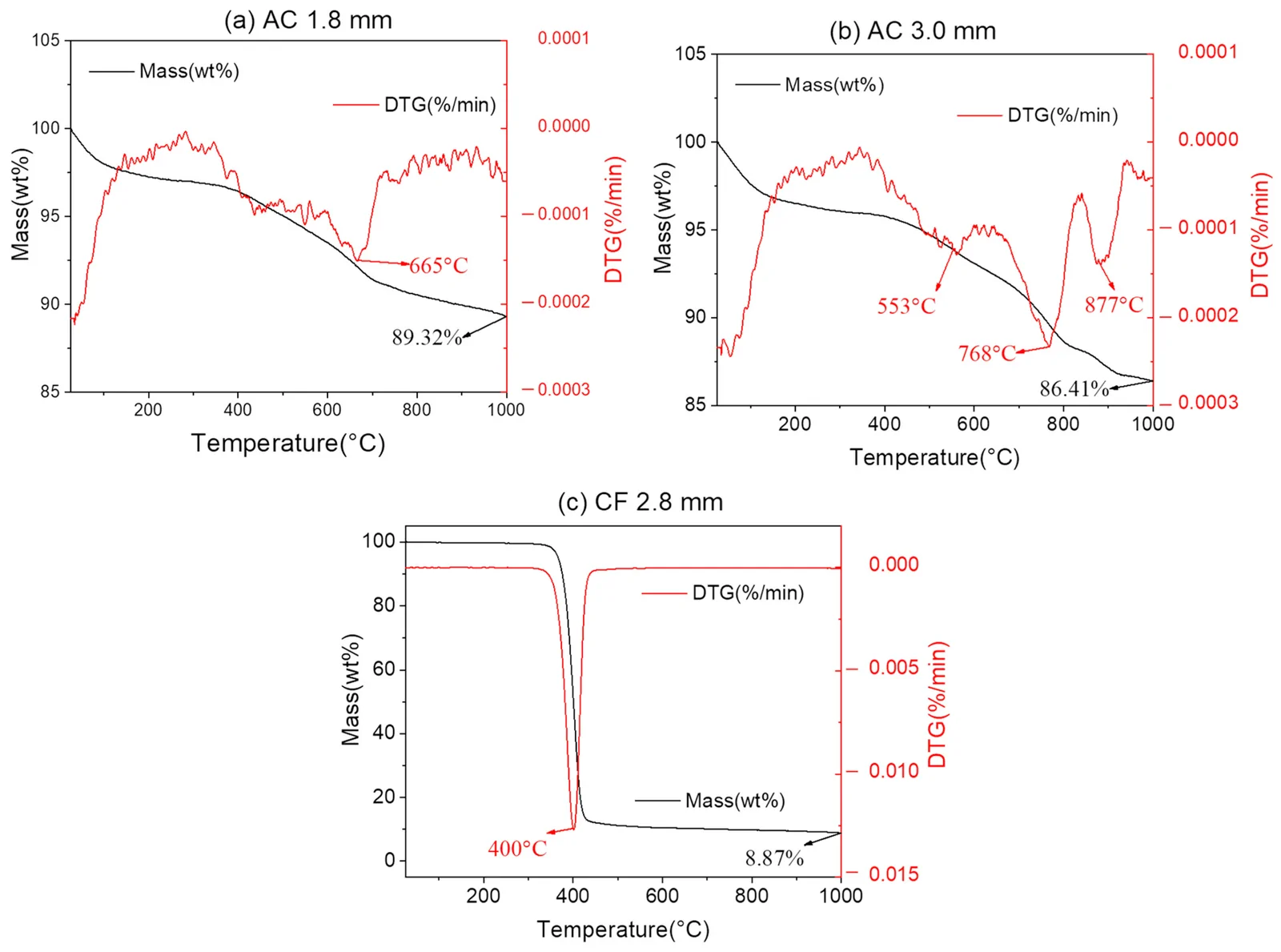
TGA (black) and derivative thermogravimetric (DTG, red) curves as a function of temperature (°C) for (**a**) AC 1.8 mm, (**b**) AC 3.0 mm, and (**c**) CF 2.8 mm. The left y-axis represents mass (wt%) and the right y-axis represents DTG (%/min). The AC samples (**a**,**b**) show multi-stage thermal decomposition leaving significant residual masses of 89.32% and 86.41% at 1000 °C, whereas the CF sample (**c**) exhibits rapid mass loss starting around 400 °C with a final residue of 8.87%.

The thermal characterizations of AC 1.8 mm (activated carbon ball of diameter 1.8 mm), AC 3.0 mm (activated carbon ball of diameter 3.0 mm) and CF 2.8 mm (carbon fibrous ball of diameter 2.8 mm) were determined through TGA performed over a temperature of 25~1000 °C. Figure 5 depicts the TGA graphs of the (a) AC 1.8 mm, (b) AC 3.0 mm and (c) CF 2.8 mm samples. The AC samples are mixed with binders to maintain their shape. Without the fibrous matrix, carbon material is structurally weak and cannot hold shape. Therefore, binders were added to hold the AC samples together [40]. Furthermore, these capacities are particularly used for cleaning substances from water, as fits the topic of endoscopy [41]. The compositional differences of the sample materials are reflected in the TGA results.

The TGA curve for AC 1.8 mm (Figure 5a) and AC 3.0 mm (Figure 5b) shows greater thermal resistance compared to the curves for CF 2.8 mm, maintaining a higher percentage of residual weight up to the temperatures 900~950 °C, thereby reflecting a larger proportion of thermally stable binder material. In the AC 1.8 mm (Figure 5a) graph, there was an initial weight loss of 2.94% below the 200 °C range and a main weight loss event of 7.71% at 600~700 °C. For the AC 3.0 mm (Figure 5b) graph, an initial weight loss of 4.06% was determined at the below the 150 °C mark, 3.22% around 700 °C, 4.56 around 750 °C, and 1.77% at above 900 °C. The graphs had residual percentages of 89.3% and 86.1% respectively, significantly higher than that of the CF 2.8 mm sample. The multiple degradation events in the TGA curves of AC 1.8 mm and AC 3.0 mm suggest characteristics and show the binder and degradation at distinct temperature thresholds. Similar multi-step degradation behavior has been reported for activated carbons containing residual binders or heterogeneous surface functionalities, which can compromise pore uniformity and adsorption performance [40,41].

The CF samples were blended with polypropylene (PP), which is a common addition to carbon fiber for its ability to improve structural and mechanical capacities. The TGA curve for the CF 2.8 mm (Figure 5c) sample exhibited the sharpest and most significant weight loss, with a drop of 91.1% between approximately 400 and 500 °C, which is the usual decomposition temperature of polypropylene. The single degradation drop indicates a material composition of minimal inorganic or residual components and can be attributed to a drop in weight in absorbed water (~100 °C) and volatile surface compounds. This TGA curve reflects the profile of a well-uniformed carbon fiber material through its dominant degradation event. The residual mass that remains at 1000 °C was 8.87%. The single dominant degradation event and low residual mass of the CF 2.8 mm balls indicate a more homogeneous carbonaceous network. By contrast, the single dominant degradation event and low residual mass of the CF 2.8 mm balls indicate a more homogeneous carbonaceous network, which is advantageous for reproducible adsorption during repeated reprocessing cycles [42,43].

### 3.2. Surface Area and Porosity

**Figure 6:**
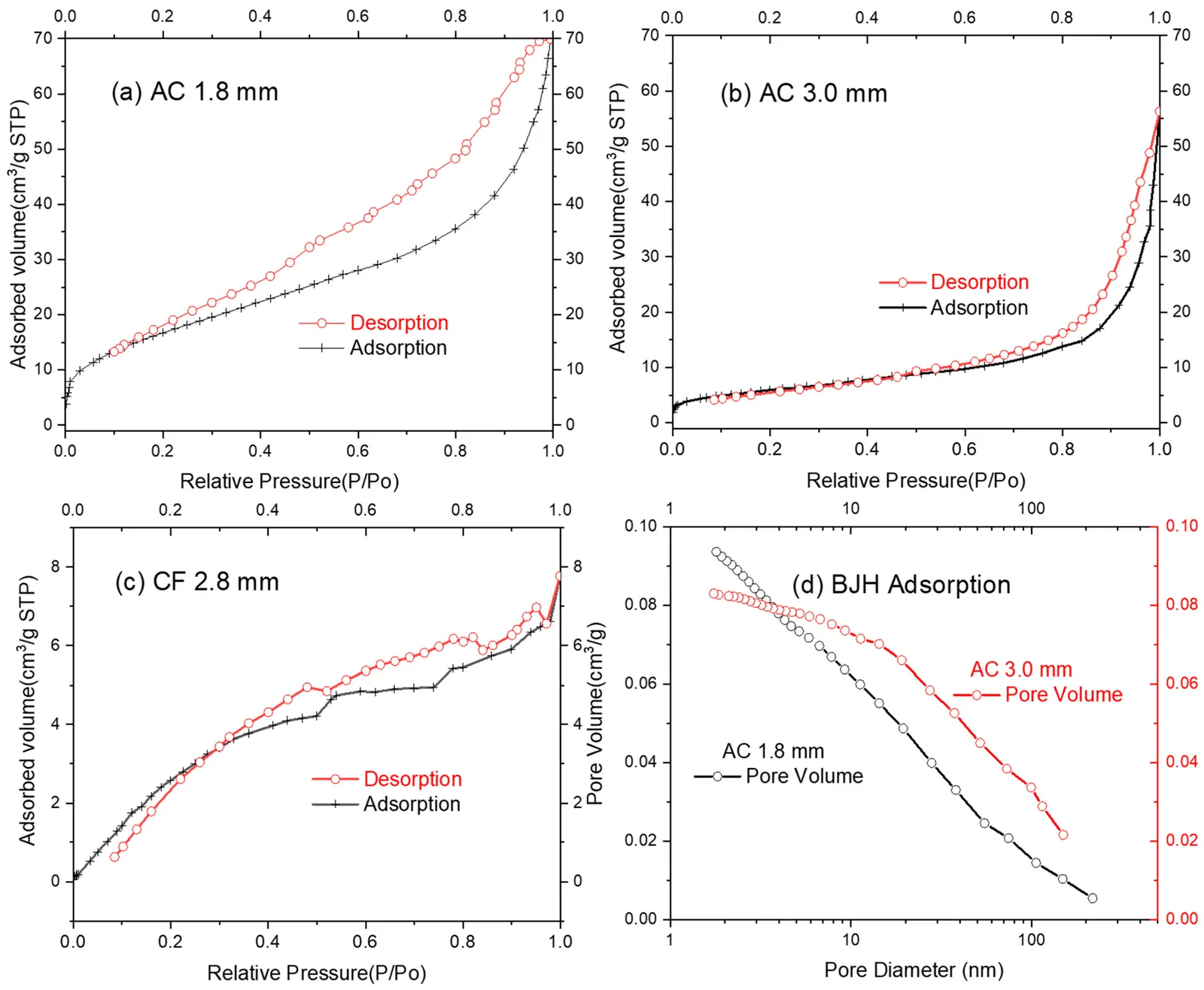
BET analysis graphs of adsorption–desorption isotherms for (**a**) AC 1.8 mm, (**b**) AC 3.0 mm and (**c**) CF 2.8 mm samples and (**d**) Barrett–Joyner–Halenda (BJH) pore size distributions. The data highlight the predominance of small pores in AC 1.8 mm and larger meso/macropores in AC 3.0 mm.

Figure 6 presents the BET adsorption–desorption analysis graphs for AC 1.8 mm, AC 3.0 mm and CF 2.8 mm samples. Figure 6a,b reflects the nitrogen adsorption–desorption isotherms of the samples. Both samples exhibited type IV-isotherms. Compared to the AC 3.0 mm sample, the AC 1.8 mm sample exhibits a significantly larger overall surface area and pore volume, despite having a smaller average pore size. Specifically, the BET surface area of AC 1.8 mm is measured at 63.00 m^2^/g with a total pore volume of 0.108 cm^3^/g. This indicates that its surface area is approximately three times larger and its pore volume is greater than those of the AC 3.0 mm sample (surface area of 21.65 m^2^/g and pore volume of 0.087 cm^3^/g). Conversely, the adsorption average pore width is larger for AC 3.0 mm (16.09 nm) than for AC 1.8 mm (6.87 nm), demonstrating that the AC 1.8 mm sample secures its expansive surface area through a densely formed, finer pore structure. The CF 2.8mm (Figure 6c) sample does not have the pore structure because of no adsorption–desorption curve. Correspondingly, Figure 6d presents the pore size distribution graph derived from the BJH method. AC 1.8mm (BJH adsorption cumulative volume of pores between 1.7000 nm and 300.0000 nm diameter: 0.093621 cm^3^/g) showed comparatively lower pore volumes throughout diameter sizes except below the 10 nm range. AC 3.0 mm (BJH adsorption cumulative volume of pores between 1.7000 nm and 300.0000 nm diameter: 0.082980 cm^3^/g) samples, on the other hand, showed larger pore volumes at larger pore diameters. Larger pore volumes in the AC 3.0mm samples may allow for greater molecular diffusion while the greater density of microporous structures in the AC 1.8mm samples can facilitate superior adsorption capabilities. Higher micropore volume generally correlates with greater adsorption capacity, whereas larger mesopores facilitate diffusion and mass transfer, especially for bulky species. The combination of a more microporous AC 1.8 mm ball and a more mesoporous AC 3.0 mm ball therefore provides a complementary pore network that can support both rapid uptake and efficient transport of endoscopic residues.

### 3.3. Morphology and Structural Features

**Figure 7:**
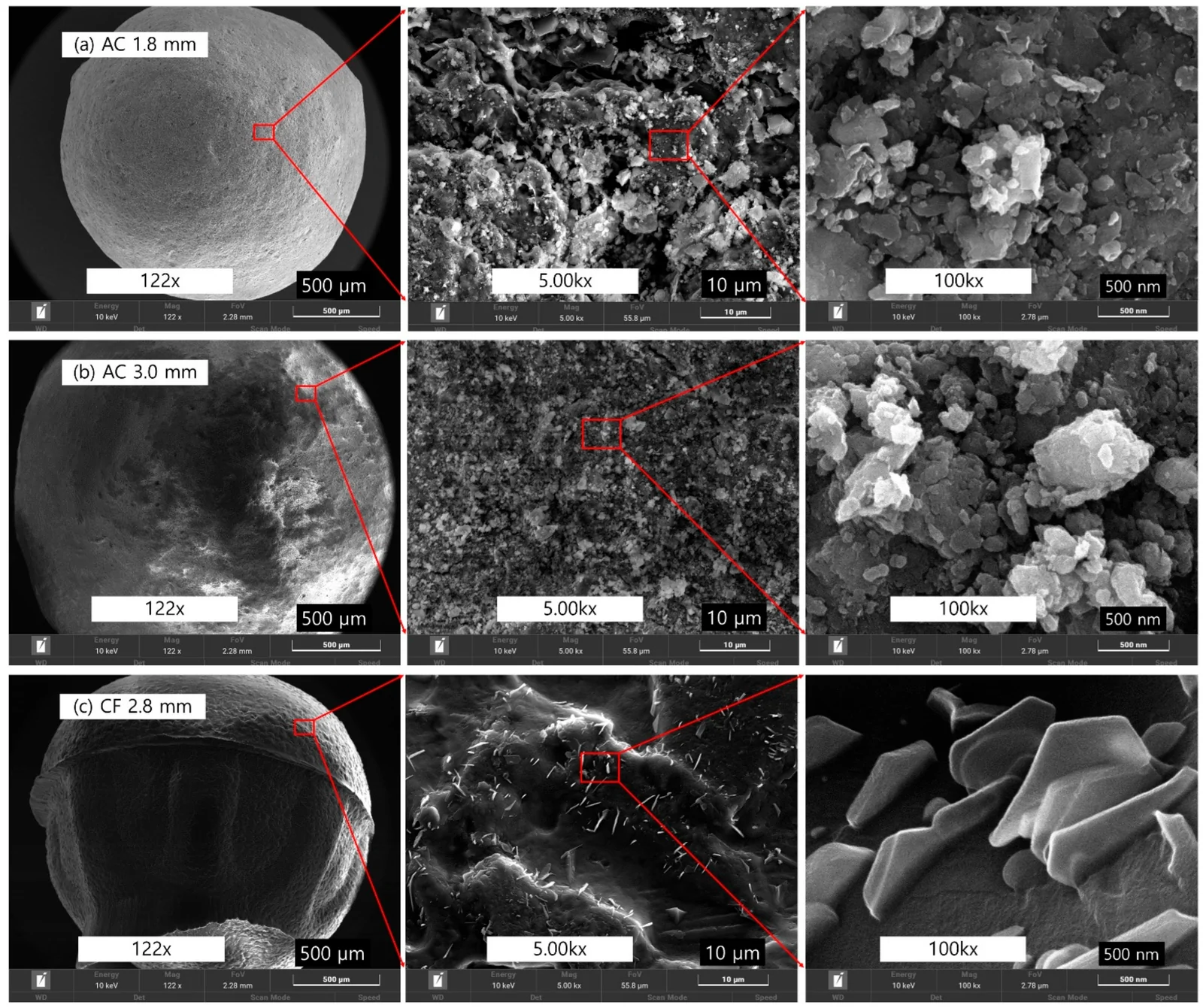
SEM images of (**a**) AC 1.8 mm, (**b**) AC 3.0 mm, and (**c**) CF 2.8 mm carbon balls. The panels from left to right display progressively higher magnifications, illustrating the overall spherical structure (**left**, 122×), intermediate surface morphology (**center**, 5000×), and high-resolution nanoscale microstructure (**right**, 100,000×). Morphologically, the AC spheres display granular and textured surfaces with jagged layers, whereas the CF spheres exhibit smoother, plate-like microstructures.

Figure 7 reinforces these morphological distinctions. Images of (a), (b), and (c) on the left are photographs magnified 122 times, while the middle and right panels are magnified 5000 and 100,000 times, respectively. AC 1.8 mm (Figure 7a) and AC 3.0 mm (Figure 7b) display granular and textured regions of microstructure. By contrast, CF 2.8 mm (Figure 7c) shows smooth microstructures that are elongated and plate-like. Figure 7 suggests a greater surface area for the AC 1.8 mm and AC 3.0 mm samples as compared to the CF 2.8 mm sample, consistent with the BET analysis of Figure 6. However, the smooth and interconnected characteristics of the CF 2.8 sample likely contributes to a greater vortex-like scouring—a measure that is not of total surface area but of the accessibility of its surface area. In this sense, higher surface area is not implicative of higher removal efficiency by activated carbons, especially for large adsorbate molecules [44]. In the CF 2.8 mm sample, there is a greater degree of plate-like accessibility as the structure is more interconnected and radially exposed. The AC samples, however, show a jaggedly layered structure in both AC 1.8 mm and AC 3.0 mm samples of the SEM images, reflecting that internal pores may be to access by adsorbate molecules [43]. The open geometry given by these differences in vortex-effective motion accessibility also suggests residual removal through the CF 2.8 mm samples and the adsorptive surface of adsorbates through the AC 1.8 and 3.0 mm samples. The greater effective surface area given by the structure, using both AC and CF samples, implies faster penetration of fluid and more rapid contaminant uptake. Endoscope biofilm studies have highlighted that rough, irregular surfaces favor initial bacterial adhesion and persistent colonization. The smoother, plate-like CF surface is expected to resist deep entrapment of soil and biofilm fragments, instead promoting transient collision and detachment, while the more rugged AC surfaces provide abundant adsorption sites for captured debris and dissolved contaminants.

### 3.4. Elemental Analysis Using EDS

**Figure 8:**
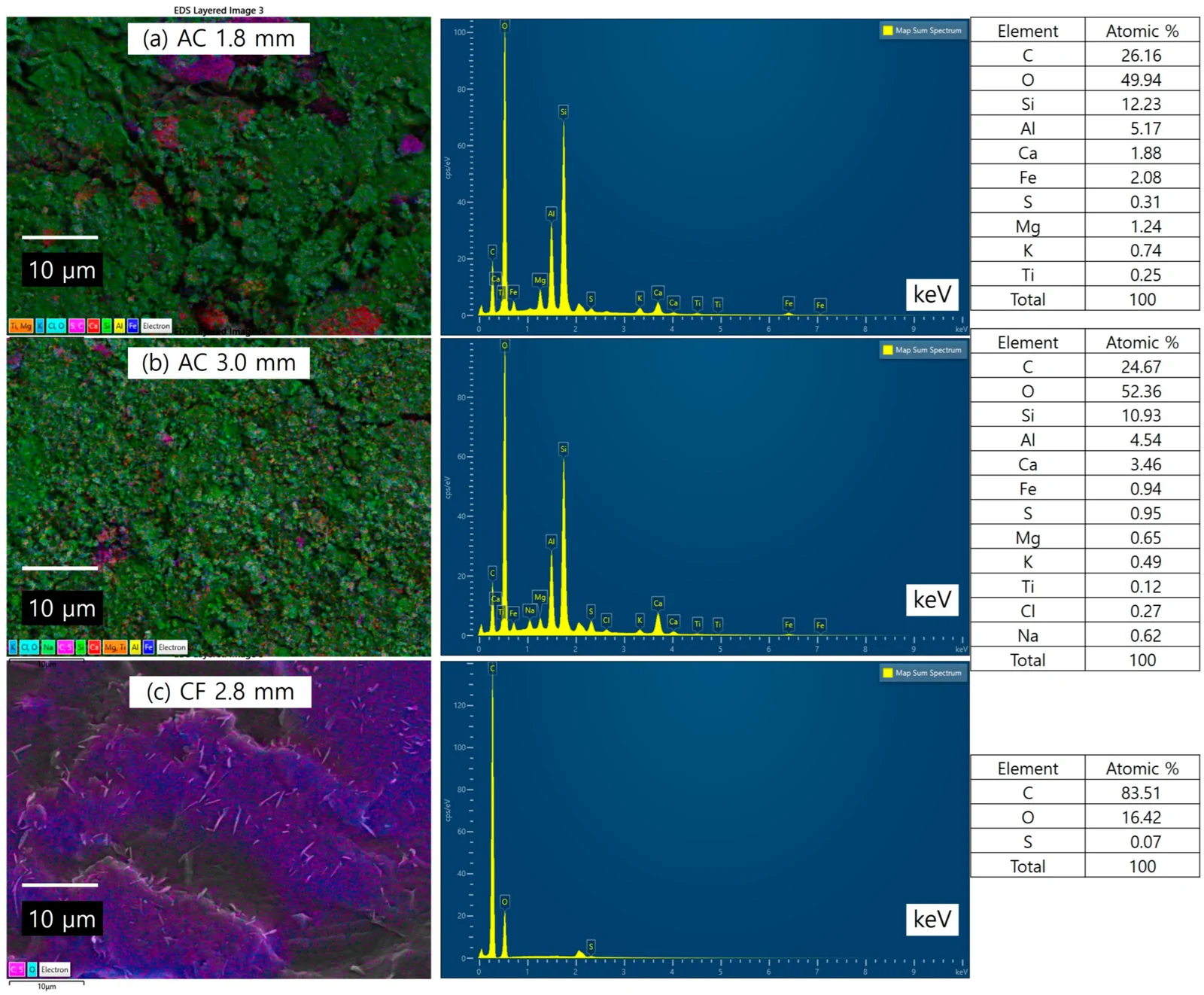
Elemental analysis of (**a**) AC 1.8 mm, (**b**) AC 3.0 mm, and (**c**) CF 2.8 mm samples. The panels display the EDS layered elemental maps (**left**), the corresponding EDS spectra showing energy peaks for identified elements (**center**), and tables detailing the quantitative elemental composition in atomic percentage (**right**). The analysis reveals that AC spheres are rich in oxygen and mineral elements like silicon, whereas CF spheres are predominantly composed of carbon (over 83 at%).

Figure 8 shows the results of energy-dispersive X-ray spectroscopy (EDS) of the identified elements obtained from (a) AC 1.8 mm, (b) AC 3.0 mm, and (c) CF 2.8 mm. On the left is the element map result of the 5000 times magnification photo in the middle of Figure 6. In the middle of Figure 8 is an X-ray peak graph of the unique elements according to energy (keV), and on the right is the atomic % of the analyzed elements. Figure 8a AC 1.8 mm and (b) AC 3.0 mm show ~25% carbon and ~50% oxygen. It can be seen that Si is present at ~11% and Al is present at 5%. It was confirmed that metals such as Ca, Fe, Mg, K, and Ti are present at approximately ~9%, unlike in the case of AC 2.8 mm. As shown in the TGA results in Figure 4, for AC 1.8 and AC 3.0, the metal components contained in the binder were present because the binder contained silicon and was mixed with activated carbon. Regarding S, which appeared in all three samples, and Cl and Na, which appeared only in AC 3.0, it is believed that impurities were present during the raw material procurement process. The substantial Si, Al and metal contents in AC 1.8 mm and AC 3.0 mm are consistent with binder-rich activated carbons reported in the environmental adsorption literature. Such inorganic phases are thermally stable (as reflected in Figure 5a,b) and can alter surface charge and hydrophilicity, potentially influencing the adsorption of ionic species present in processing solutions. Figure 8c CF 2.8 mm shows 84% carbon and 16% oxygen elements. As in the TGA result of Figure 5c, this is because CF 2.8 was molded by mixing PP and carbon fiber.

### 3.5. Morphology and Elemental Features After Immersed Balls (IBs) in Aqueous Tomato Powder Solution

Despite repeated reprocessing of flexible endoscopes, residual organic matter and viable bacteria can gradually accumulate along inner channels, forming persistent biological debris [45]. Although clinical contamination is highly complex—typically comprising viscoelastic mixtures of mucin, proteins, blood, lipids, and mature biofilms—an aqueous tomato powder solution was selected for this initial proof-of-concept evaluation to mimic the viscosity and adhesive organic load of such accumulated residue [45]. Furthermore, as a non-hazardous particulate simulant, its high optical contrast and discrete particulate nature enabled clear visual and microscopic tracking of the carbon balls’ mechanical detachment and adsorption dynamics. Therefore, this simplified model was utilized to isolate and validate the fundamental hydrodynamic washing action of the carbon balls prior to introducing the rheological complexities of highly cohesive clinical bioburden, rather than to assert direct clinical relevance as a contamination model.

**Figure 9:**
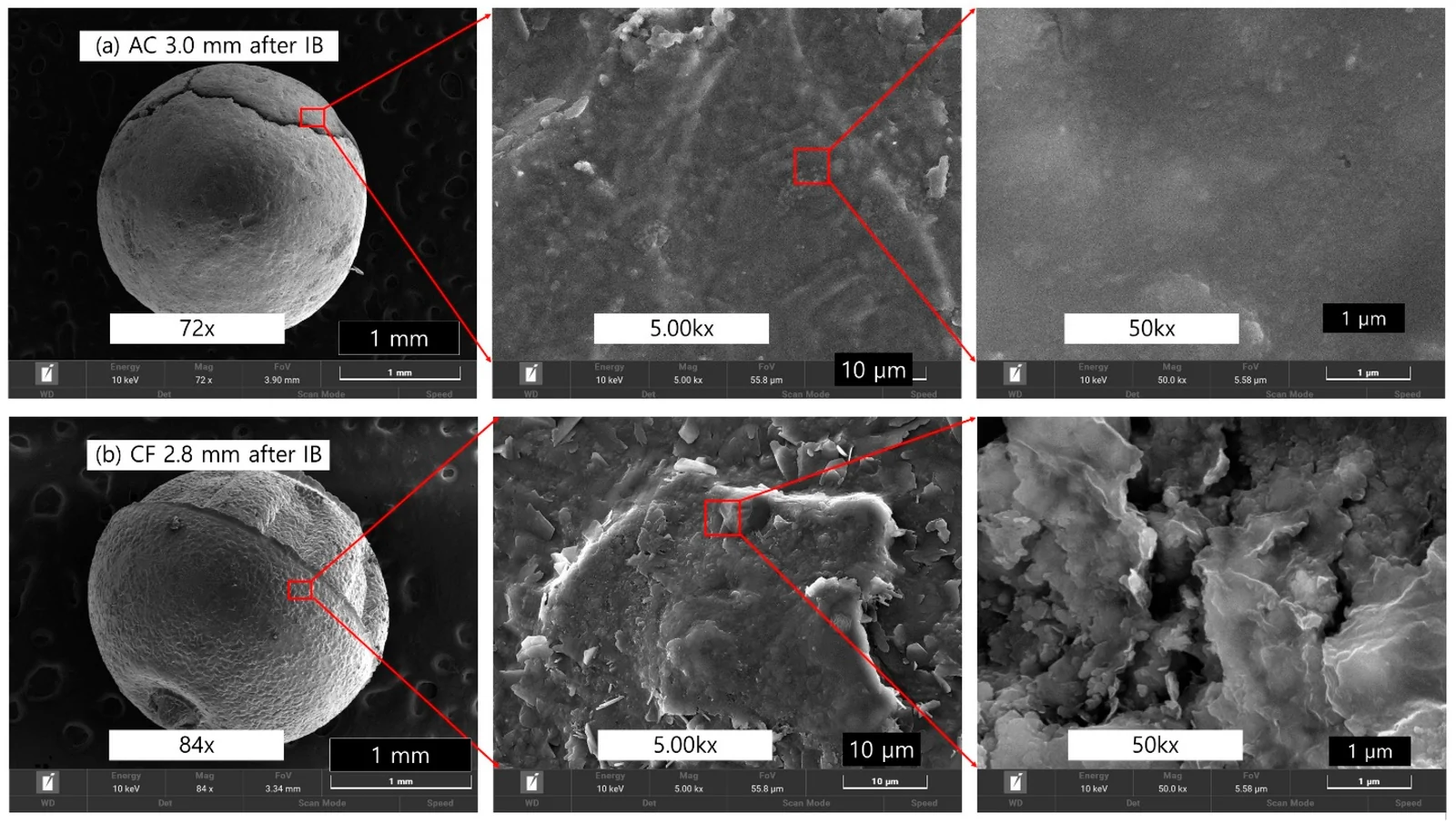
SEM images of (**a**) AC 3.0 mm and (**b**) CF 2.8 mm carbon balls after the immersed ball (IB) process. The panels from left to right display progressively higher magnifications, illustrating the macroscopic view of the entire ball (**left**), the intermediate surface morphology (**center**, 5000×), and the high-resolution microstructure (**right**, 50,000×) of the corresponding highlighted regions.

Following this rationale, the balls of AC 3.0 and CF 2.8 were immersed in a tomato powder solution prepared with deionized water and subsequently passed through a 3.5 mm tube. Figure 9 shows the microstructure of (a) AC 3.0 and (b) CF 2.8 after passing the tube ten times. While both samples showed no morphological changes, indicating no clumping, the microscopic surface topography showed changesAs shown in Figure 9a, for AC 3.0,impurities inside the endoscope were confirmed to have wrapped around the surface of the ball. In Figure 9b, for CF 2.8,the plate-like shape of the carbon fibers was changed to a smooth shape, confirming the protrusions that interacted with tomato powder. Furthermore, tomato powder was confirmed to be attached to the surface. This suggests that adsorption occurs first when the tomato powder on the activated carbon comes into contact with it.

This suggests that the simultaneous use of AC and CF balls can repel and adsorb impurities, resulting in more effective cleaning. The observed smoothing of CF protrusions and the coating of AC 3.0 mm surfaces with adherent material after a single cleaning cycle support a mechanistic model in which CF balls first dislodge soil from the channel wall, followed by adsorption and “wrapping” of this material onto AC balls, thereby mimicking a two-step detachment–capture process within the lumen.

**Figure 10:**
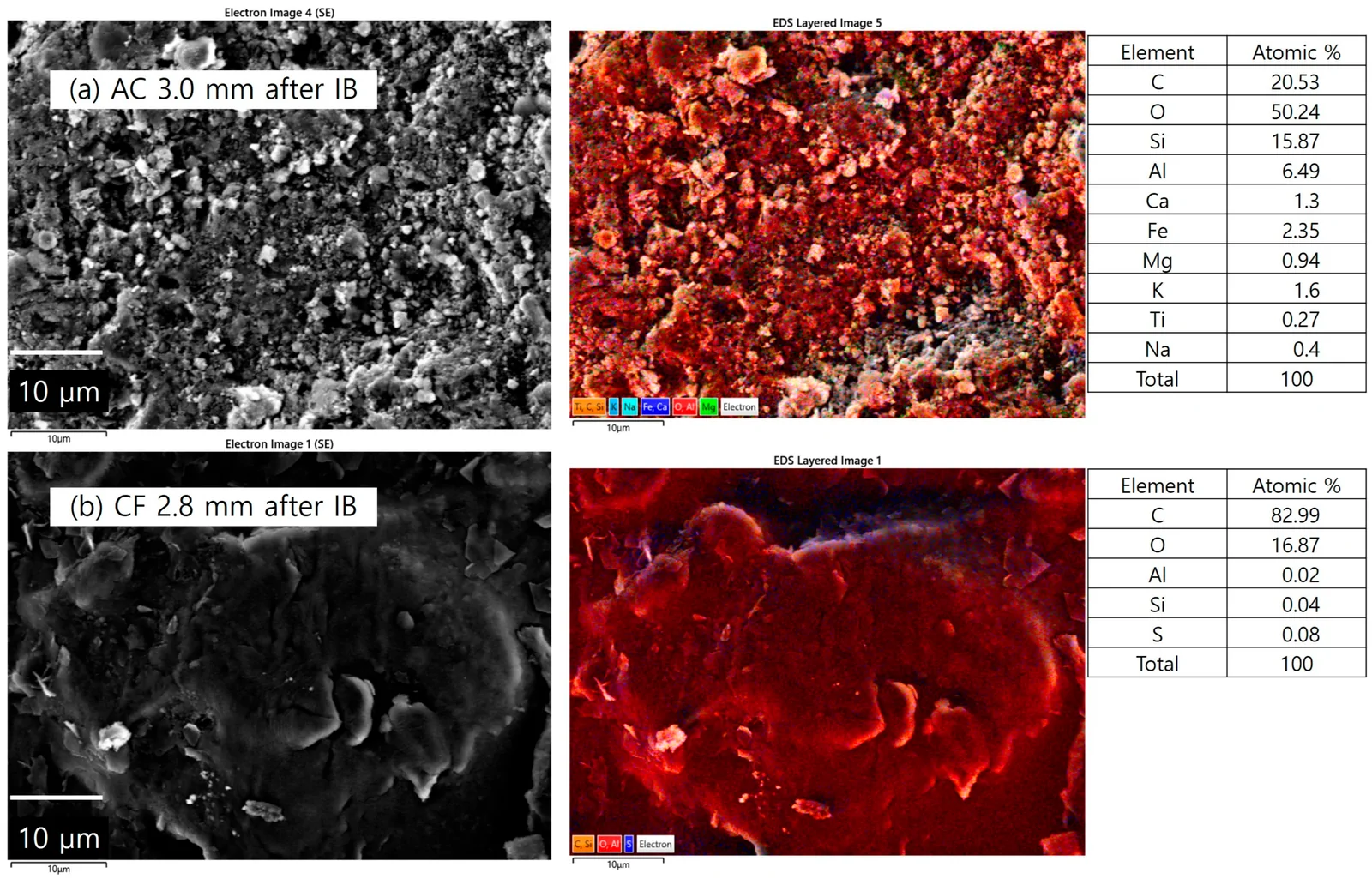
Surface morphology and elemental analysis of (**a**) AC 3.0 mm and (**b**) CF 2.8 mm samples after the immersed ball (IB) process. The panels display the SEM image showing surface topography (**left**), the EDS layered map showing elemental distribution (**center**), and a table detailing the quantitative elemental composition in atomic percentage (**right**).

The left images of Figure 10 present a 5000× magnification image of the region not covered by tomato powder in the sample shown in Figure 9, while the center images display the corresponding elemental map. The right tables summarize the atomic contents (%) of the analyzed elements. AC 3.0 mm (Figure 10a) exhibited ~20% carbon and ~50% oxygen, with additional elements including ~15% Si, 7% Al, and ~8% combined contributions from Ca, Fe, Mg, K, and Ti. These findings, consistent with the EDS results in Figure 8b, indicate that while the surface of the AC sphere partially adsorbs components from the aqueous solution, it does not undergo chemical reaction or structural deformation. In contrast, for CF 2.8 mm (Figure 10b), the composition was 84% carbon and 16% oxygen, consistent with the EDS results in Figure 8c. This confirms that the surface of the sphere does not undergo chemical reaction or deformation in the aqueous solution.

### 3.6. Cleaning Performance of AC and CF Spheres Immersed in Aqueous DI Water Solution

In Korean hospitals, endoscope disinfection and reprocessing procedures consist of six steps: pre-washing, cleaning, disinfection, rinsing, drying, and storage [46]. This study was conducted in collaboration with the Seegene Medical Foundation, FITI Testing & Research Institute (FITI), and Korea Testing & Research Institute (KTR). Prior to reprocessing, endoscope working channels were inoculated to establish a quantitative baseline bioburden of >10^6^ CFU/mL. To ensure statistical reliability and high experimental confidence and eliminate single-laboratory bias, identical post-cleaning samples from independent channel evaluations were tested across these three accredited testing laboratories. Across all repeated and cross-validated tests, the microbiological results consistently showed a residual microbial load of <10 CFU/mL. These repeated multi-center evaluations confirm the statistical significance and high decontamination effectiveness of the carbon ball system relative to the pre-cleaning baseline. The test samples consisted of dedicated post-cleaning sampling effluent obtained by passing an additional sterile water flush through the gastroscope channel immediately after CF ball cleaning, specifically designed to detect and recover any residual bacteria remaining on the internal lumen. The samples were analyzed under identical conditions (sterile container collection, room temperature transport, and culture within 24 h) at each of the three institutions. All tests adhered to the ISO 15883-5 (Endoscope Cleaning Validation) standard, and bacterial and fungal counts were quantitatively evaluated in CFU/mL. The tests were performed under the standard cleaning program conditions of the endoscope washer (flow rate 0.5 L/min, cleaning solution temperature 40 °C). The cleaning process proceeded as follows:

(i) Preparation of cleaning solution: Enzymatic detergent was diluted 1:100 and injected into the cleaning tank.
(ii) Introduction of CF sphere balls: Two ribbed sphere balls, approximately 2.7 mm in diameter, were inserted into the endoscope channel inlet.
(iii) Circulation washing: Washing water was circulated once through the channel with the sphere balls to induce mechanical contact and fluid turbulence.
(iv) Collection of post-cleaning sampling effluent: Following the carbon ball cleaning process, an additional volume of sterile water was aspirated through the gastroscope channel to sample residual bioburden from the internal wall. The discharged sampling fluid was collected under aseptic conditions and immediately transported to the accredited testing laboratories for microbiological examination.
(v) Culture analysis: Microbial colonies were counted after culturing at 30–35 °C for 48–72 h according to the standard testing method of each laboratory.

The results reported by the three laboratories of A, B, and C were as follows:

A. Seegene Medical Foundation: the general bacterial culture result was 0 CFU/mL, meaning no bacteria were detected in the culture. B. FITI Testing & Research Institute: both general bacterial and fungal counts were confirmed to be <10 CFU/mL, below the detection limit. C. KTR (Korea Testing & Research Institute for Chemical Industry): both total aerobic microorganism counts and total fungal counts were <10 CFU/mL, both below the detection limit.

In other words, bacteria and fungi were either not detected or were statistically insignificant (below the detection limit) in all three institutions. This result can be interpreted as a level of culture sterility, which corresponds to the “undetectable level of residual contamination after cleaning” defined by the ISO 15883-5 standard.

Although this study primarily focuses on the conceptual and structural design of the biomimetic brush without conducting direct empirical control experiments (e.g., positive/negative controls, conventional brush comparisons, or before–after bacterial counts), the proposed efficacy is robustly supported by established principles in the recent literature [3,47,48,49,50]. First, regarding the absence of before–after bacterial counts, foundational guidelines on endoscope reprocessing [3,47] establish that meticulous mechanical cleaning inherently reduces the microbial burden by a factor of 3 to 6 logs (10^3^ to 10^6^). This equates to a 99.9% to 99.9999% reduction in bioburden even before high-level chemical disinfection is applied. Because this mechanical reduction rate is a scientifically validated standard, our design aims to theoretically optimize this physical friction phase rather than duplicating standard microbial counting. Furthermore, recent clinical practices widely rely on rapid cleaning tests, such as ATP fluorescence, to proxy microbial removal, validating that physical design improvements directly correlate with reduced bioburden.

Second, addressing the comparison with conventional brushes, recent prospective studies [48] demonstrate that structural and material innovations inherently outperform traditional single-headed nylon brushes. For example, brushes incorporating novel materials, such as non-woven fabrics in a double-headed structure, achieved significantly higher cleaning efficacy compared to conventional models. This superior efficacy was quantitatively proven by greater ATP reduction and higher bacterial removal rates. Additionally, controlled experimental studies evaluating surface extraction efficiency have shown that advanced materials (e.g., tissue non-tissue wipes/ball brush made of a silicon ball wrapped in microfibers) can extract up to 93% of Bacillus atrophies’ spores [49,50]. This extraction efficiency is vastly superior to the 52% efficiency observed with traditional cellulose materials, which function as a theoretical positive control for optimal material performance. By integrating these proven material and structural concepts, our biomimetic design provides a theoretically superior cleaning mechanism without the immediate need for comparative control trials.

Finally, improving mechanical cleaning efficiency fundamentally aligns with the critical clinical shift towards sustainable “green endoscopy” [47]. Redesigning endoscopic accessories to maximize first-pass mechanical cleaning efficacy can prevent reprocessing failures and significantly reduce the environmental footprint associated with redundant washing cycles, prolonged chemical usage, and excessive disposable waste. In conclusion, while direct in vitro control experiments were omitted in this developmental phase, the biomimetic parameters proposed in this study are theoretically grounded in validated microbiological metrics, material science, and current clinical standards.

### 3.7. Flow and Pressure Fields Around Carbon Balls in Endoscope Channels

To directly link carbon ball geometry to mechanical cleaning performance, the numerically simulated pressure and velocity fields in the flexible endoscope channel were analyzed.

#### 3.7.1. Pressure Distribution and Driving Force

**Figure 11:**
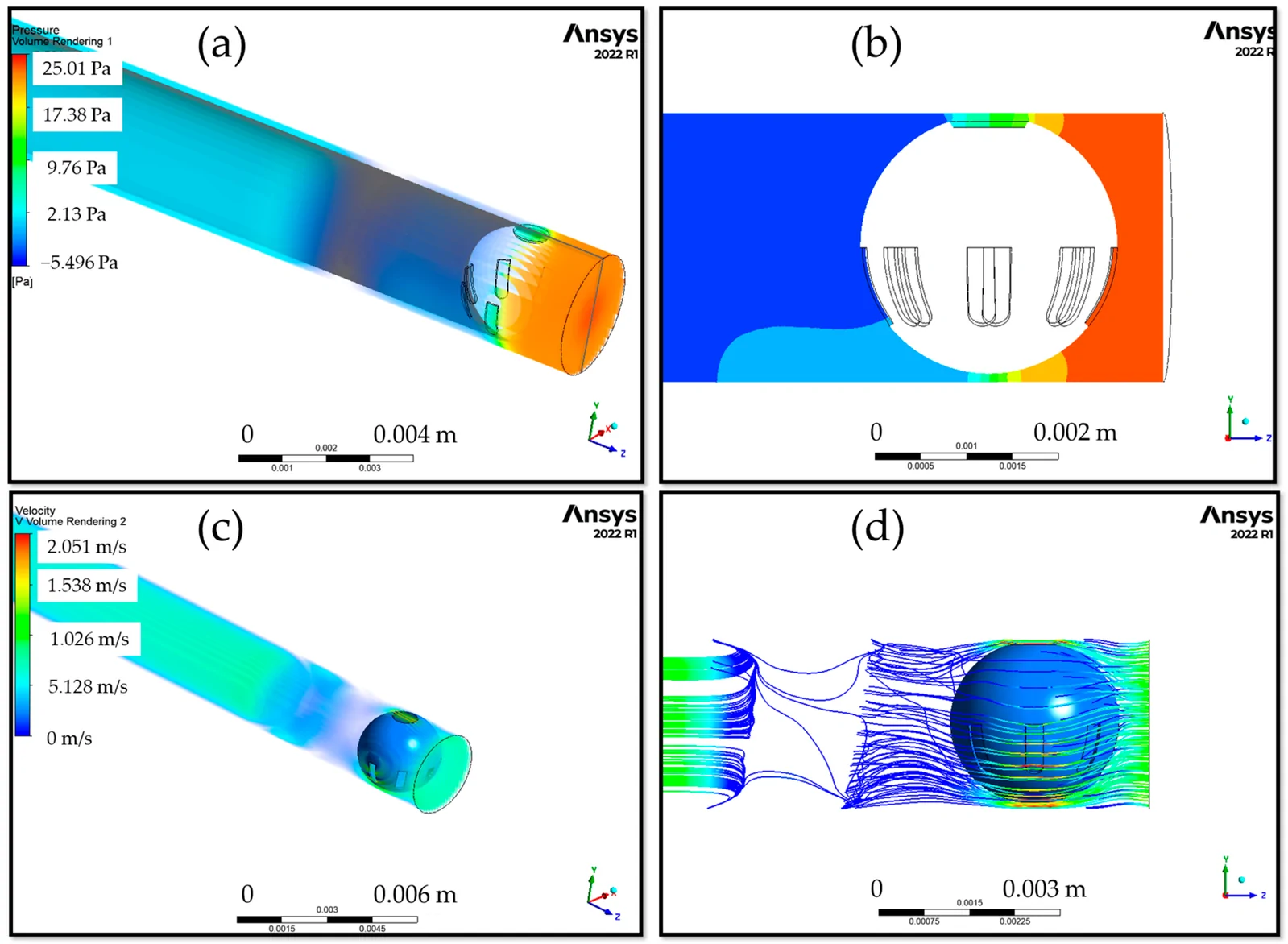
Flow characteristic analysis results around the ball: (**a**) isometric view of pressure distribution, (**b**) front view of pressure distribution, (**c**) isometric view of velocity distribution, and (**d**) front view of velocity distribution with streamlines. A maximum pressure of 25.0 Pa and a minimum of −5.4 Pa were recorded, while the pressure acting on the narrow gaps at the top and bottom of the ball was approximately 9.8 Pa. A maximum flow velocity of 2.0 m/s was observed through the narrow passages at the top and bottom of the ball, with an average flow velocity inside the pipe calculated to be 0.5 m/s.

Figure 11a,b present the pressure distribution around the carbon ball and along the inner wall of the flexible endoscope channel ((a) isometric view and (b) front view). High-pressure regions (approximately +25 Pa, shown in red) were observed on the upstream side of the carbon ball and other internal structures, reflecting stagnation of the cleaning solution as it impinges on the ball. In contrast, low-pressure regions (approximately −5.4 Pa, shown in blue) formed downstream of the ball, where flow acceleration and vortex formation induced a local pressure drop. The resulting pressure difference across the ball was approximately ΔP ≈ 30 Pa, which is directly related to the hydrodynamic drag acting on the ball. This pressure-driven force is responsible for translating and rotating the carbon ball along the channel, thereby promoting repeated contact with the channel wall and enhancing mechanical cleaning of residual bioburden. Importantly, the pressure distribution on the channel wall also indicates zones where high wall shear stresses are expected, particularly near the narrow gap between the ball and the wall. These zones coincide with locations where mechanical detachment of biofilm and organic residue is most likely to occur, supporting the experimental observation that CF balls induce strong vortex flow and wall interaction without damaging the channel surface.

#### 3.7.2. Velocity Distribution and Vortex Formation

Figure 11c,d show the velocity magnitude distribution around the carbon ball of isometric view and front view. High-velocity regions (approximately 2.0 m/s, shown in red) were observed where the fluid was forced through the narrow clearance between the ball and the wall and around the sides of the ball. Low-velocity regions (near 0 m/s, shown in blue) appeared in the wake behind the ball and in boundary layers near the wall, corresponding to stagnation and recirculation zones. The overall flow pattern was characteristic of flow around a spherical body: fluid decelerated in front of the ball, accelerated along the sides, and formed vortices downstream. The velocity difference of approximately ΔV ≈ 2 m/s between high-speed and low-speed zones was closely linked to the pressure distribution through the Bernoulli relationship and translated into significant wall shear stresses along the channel wall. High-velocity, low-pressure regions adjacent to the wall are particularly favorable for mechanical cleaning, as they generate large shear forces that can dislodge adherent bioburden. This numerical finding is consistent with the proposed design of ribbed CF balls, which aim to induce micro-rotational and swirling motion and thereby create vortex-like flow regimes along the channel walls.

#### 3.7.3. Correlation with Experimental Cleaning Performance

The numerical results support the experimental cleaning tests performed with CF balls under standardized endoscope reprocessing conditions. In the hospital-based validation, wash water collected after cleaning gastroscope channels with CF balls showed bacterial and fungal counts below the detection limit (≤10 CFU/mL), corresponding to an “undetectable level of residual contamination” according to ISO 15883-5. Combining these microbiological results with the CFD analysis suggests that the ribbed CF balls generate sufficient wall shear stress and vortex-induced scouring to detach residual contaminants, while the AC balls provide complementary adsorption of detached debris and dissolved organic species. Thus, the numerical model offers a mechanistic explanation for the experimentally observed sterility-level cleaning performance when carbon balls are integrated into standard endoscope reprocessing workflows.

### 3.8. Implications for Endoscope Cleaning Practice

The combined thermal, structural, porosity, and elemental analyses indicate that AC and CF balls offer several advantages for use in endoscope channels. The highly activated, compositionally uniform fibrous matrix and ribbed outer surface support both adsorption-driven and mechanical cleaning mechanisms, while the ball size is compatible with commonly used working channels. Carbon balls, although potentially useful as low-cost, structurally robust alternatives, display more heterogeneous compositions and morphologies that may limit effective surface accessibility, particularly for larger or more complex contaminants.

By reducing reliance on rigid bristle brushes, carbon balls could help mitigate channel wall damage and associated biofilm formation, address operator-dependent variability in manual cleaning, and improve the consistency of reprocessing outcomes. Future work should include systematic microbiological efficacy studies, flow dynamics analysis within instrument channels, durability testing over repeated cleaning cycles, and evaluation of compatibility with different endoscope models and reprocessing protocols. Meta-analyses and national surveillance efforts have consistently reported contamination rates of approximately 10–20% in patient-ready endoscopes, with links to clinically significant infectious outbreaks. Integrating AC and CF carbon balls into the manual cleaning step could therefore provide a materials-based countermeasure that directly targets the critical gap between visual channel cleanliness and microbiological safety.

The rigorous suppression of microbial bioburden achieved by our brush-free alternative biomimetic approach holds substantial clinical relevance. Gastrointestinal endoscopy-associated infections remain a persistent challenge, primarily driven by bacterial pathogens such as *Pseudomonas aeruginosa* and *Enterobacteriaceae* that colonize intra-channel surfaces [51]. Recent large-scale nationwide surveillance data emphasize that standard automated and manual reprocessing protocols frequently leave detectable microbial contamination in clinical practice, underscoring an urgent demand for enhanced cleaning efficiency [11]. In this context, achieving complete bioburden clearance (<10 CFU/mL or 0 CFU/mL) via our swirling carbon ball mechanism directly addresses these clinical shortcomings. Furthermore, the sampling and quantification framework employed in this study aligns with validated microbiological monitoring protocols, which have been shown to effectively capture residual intra-channel culturable flora compared to conventional methods [13]. While these controlled challenge experiments demonstrate robust baseline efficacy, future clinical trials evaluating complex, multi-species clinical biofilms will further delineate the translational limits of this technology.

## 4. Discussion

Inadequate cleaning of flexible endoscope working channels remains a persistent challenge in gastrointestinal endoscopy, contributing to healthcare-associated infections despite standardized reprocessing guidelines. Traditional bristle brushing, while effective when performed meticulously, is operator-dependent and associated with channel wall damage that promotes biofilm formation and residual microbial entrapment. The carbon spheres developed in this study address these limitations by combining gentle mechanical action—through ribbed CF geometry and vortex-inducing motion—with adsorption capacity from AC’s porous structure, offering a promising brush-free alternative.

The thermal, morphological, and elemental characterizations reveal complementary strengths between CF and AC balls that support a synergistic cleaning mechanism. CF 2.8 mm balls demonstrated superior compositional uniformity (single-step 91.1% mass loss at 400–500 °C, 8.9% residual mass), plate-like microstructures, and carbon–oxygen dominance, facilitating effective collision-based detachment without structural degradation during reprocessing cycles. In contrast, AC 1.8 and 3.0 mm balls exhibited binder-derived heterogeneity (multi-step degradation, 86–89% residual mass) but complementary pore architectures. The chemical rationale behind the 91:9 wt% elastomer-to-carbon ratio and its rapid capture mechanism can be explained through a biomimetic analogy to natural seed mucilage (Figure 2). Recent studies demonstrate that seed mucilage relies on a highly dominant polymeric matrix embedding a remarkably small fraction of natural cellulosic nanofibers to achieve exceptional hydration, adhesion, and ecological anchorage against fluid flow [21,22,23,24,25,26]. Similarly, our carbon composite balls do not rely on bulk solid carbon mass for adsorption. Instead, the 91 wt% elastomeric binder serves as a macroscopic structural scaffold—analogous to the seed mucilage envelope—facilitating extremely rapid fluid interaction and providing the necessary elastic compliance for safe navigation. Within this dominant matrix, the 9 wt% functional carbon fillers act as the active binding nodes, directly mimicking the functional cellulosic nanofibers in natural mucilage [23,25]. Based on this biomimetic architecture, we hypothesize that the exposed carbon network instantaneously adsorbs organic matter upon fluid contact, prior to macroscopic physical motion by ribbed structures to generate vortex-like scouring. However, while morphological observations suggest rapid bioburden capture, qualitative imaging alone is insufficient to definitively conclude this sequence. To validate the adsorption kinetics, future studies will incorporate real-time mass gain analysis and time-resolved spectroscopic techniques to quantitatively track molecular binding sequences during dynamic fluid transport.

To ensure statistical reliability, high experimental confidence, and eliminate single-laboratory bias, washer-disinfector validation using the carbon balls was conducted in collaboration with the Seegene Medical Foundation, FITI Testing & Research Institute (FITI), and Korea Testing & Research Institute (KTR). Across all repeated evaluations, identical post-cleaning samples consistently demonstrated a residual microbial load of <10 CFU/mL (or 0 CFU/mL), successfully meeting the ISO 15883-5 criteria. This multi-center cross-validation rigorously confirms the highly reproducible and effective decontamination performance of the carbon ball system. These findings align with prior reports linking channel damage to biofilm persistence and underscore the need for gentler tools. Unlike brushes, carbon spheres avoid point-loading while enhancing fluid dynamics through micro-rotation and rib-induced turbulence. However, while the current study established cleaning efficacy across these independent institutions, the potential for micro-particle shedding from the hard carbon–polymer composite balls due to tight clearance friction remains a limitation. Comprehensive particle-residue and cytotoxicity evaluations are essential and will be the primary focus of our future investigations prior to clinical translation.

Despite the promising mechanical cleaning and adsorption capabilities, this study has certain methodological limitations. Specifically, we investigated surface interactions, such as adsorption with hydration, on the carbon balls using a simplified organic particulate simulant (tomato powder) for the visual and physical clearance assessments. Clinical endoscope soils are significantly more challenging, characterized by highly cohesive and adhesive mixtures of mucin, hemoglobin, lipids, and mature biofilms. Furthermore, while the post-cleaning sampling flush combined with CFD analysis provided robust proof-of-concept data for this brush-free system, direct mechanical sampling of the channel lumen was not performed to avoid confounding the hydrodynamic action of the carbon balls with brush-induced artifacts. Standardized Flush–Brush–Flush (FBF) or Flush–Swab–Flush sampling protocols, as outlined in ISO 15883-5 and ASTM F3208-20 [52], represent the benchmark for recovering adherent biofilms. Therefore, future studies will be required to validate the quantitative decontamination efficacy using clinically relevant Artificial Test Soils (ATSs) and established microbiological biofilm models, adopting standardized FBF protocols to directly quantify the physical detachment of mature biofilms from the channel surface.

Another limitation of this study is the lack of a direct microbiological comparison, including log reduction reporting, with conventional brush cleaning. However, the inherent vulnerabilities of manual cleaning are already well documented. Manual cleaning heavily relies on meticulous human effort, and slight deviations from protocols frequently leave behind microbial residuals. Consequently, previous studies have shown that at least 15% of endoscope uses result in contamination by gastrointestinal pathogens, such as *Enterobacter cloacae*, *Escherichia coli*, and *Klebsiella pneumoniae*. Because inadequate manual cleaning remains a significant cause of nosocomial infections and iatrogenic transmission, our study focused on validating the proposed method’s potential to bypass these specific human-error risks. Future studies should incorporate conventional brush cleaning as a control group to report comparative log reductions.

Finally, while our approach focuses on the physical and material-based decontamination of medical instruments using carbon composites, the broader field of microbial disinfection has recently seen remarkable advancements through innovative material designs, such as hot electron-assisted photothermal catalysts for solar-driven environmental remediation [53]. Integration of advanced material strategies into automated washers as a standardized adjunct could bridge the gap between visual cleanliness and microbiological safety, transforming endoscope reprocessing from an operator-dependent art to a reproducible science.

## 5. Conclusions

We developed and characterized activated carbon (AC) and carbon fiber (CF) carbon balls as adjunctive cleaning elements for gastrointestinal endoscope channels and further evaluated their hydrodynamic behavior using numerical analysis in a representative flexible channel model. Thermal, structural, and morphological analyses indicated that AC balls offer high surface area and heterogeneous pore structures suitable for adsorption, whereas CF balls provide a more uniform fibrous architecture and ribbed geometry optimized for mechanical interaction with the channel wall.

The ANSYS-based CFD analysis demonstrated that a carbon ball inside a 2.7 mm flexible endoscope channel generates a pressure difference of approximately 30 Pa and a velocity difference of about 2 m/s, producing high wall shear stress and vortex flow in the narrow gap between the ball and the channel wall. These flow conditions are favorable for mechanical removal of residual bioburden, particularly in regions where the shear stress exceeds the adhesion strength of the debris layer. When combined with microbiological validation showing bacterial and fungal counts at or below the detection limit after CF ball-assisted cleaning, these findings support the feasibility of using carbon balls as gentle, adsorption-enhanced mechanical cleaning elements that might complement, rather than replace, traditional bristle brushes in routine endoscope reprocessing.

Notably, the concept is further strengthened by biomimetic inspiration from mustard seed surface structures, which provide a natural model for compact geometry, surface heterogeneity, and interfacial interactions relevant to adsorption and debris removal. Future studies should refine ball geometry and material composition based on coupled experimental–numerical optimization and assess long-term durability and compatibility across different endoscope models and reprocessing protocols.

## Data Availability

The data presented in the paper are available upon request.
